# Advantages of Heterotrophic Microalgae as a Host for Phytochemicals Production

**DOI:** 10.3389/fbioe.2021.628597

**Published:** 2021-02-12

**Authors:** Surumpa Jareonsin, Chayakorn Pumas

**Affiliations:** ^1^Department of Biology, Faculty of Science, Chiang Mai University, Chiang Mai, Thailand; ^2^Research Center in Bioresources for Agriculture, Industry and Medicine, Department of Biology, Faculty of Science, Chiang Mai University, Chiang Mai, Thailand

**Keywords:** microalgae, heterotroph, phytochemical, transformation, host system

## Abstract

Currently, most commercial recombinant technologies rely on host systems. However, each host has their own benefits and drawbacks, depending on the target products. Prokaryote host is lack of post-transcriptional and post-translational mechanisms, making them unsuitable for eukaryotic productions like phytochemicals. Even there are other eukaryote hosts (e.g., transgenic animals, mammalian cell, and transgenic plants), but those hosts have some limitations, such as low yield, high cost, time consuming, virus contamination, and so on. Thus, flexible platforms and efficient methods that can produced phytochemicals are required. The use of heterotrophic microalgae as a host system is interesting because it possibly overcome those obstacles. This paper presents a comprehensive review of heterotrophic microalgal expression host including advantages of heterotrophic microalgae as a host, genetic engineering of microalgae, genetic transformation of microalgae, microalgal engineering for phytochemicals production, challenges of microalgal hosts, key market trends, and future view. Finally, this review might be a directions of the alternative microalgae host for high-value phytochemicals production in the next few years.

## Introduction

**FIGURE 1:**
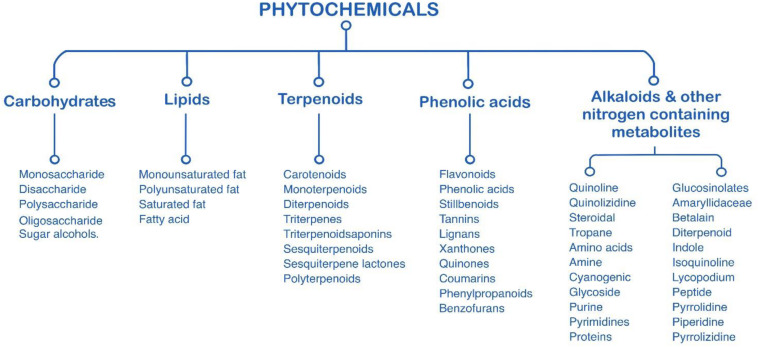
Characterization of phytochemicals (adapted from Xiao et al., 2016).

Plant chemicals or phytochemicals are chemicals that may have biological activities produced by plants. Phytochemical sources come from fruits, vegetables, whole grains, nuts, seeds, leaves, bark, flowers, and other part of plants. Bioactive phytochemicals have been extensively studied *in vitro* and *in vivo* models due to their great potential for human consumption. Generally, phytochemicals were classified into six major categories based on their chemical structures and characteristics (Figure 1) including lipids, carbohydrates, terpenoids, phenolics, alkaloids, and other nitrogen-containing compounds (Xiao et al., 2016). Similarly, microalgae are promising natural sources of various bioactive compounds, such as polysaccharide paramylon, polyunsaturated fatty acids, and pigments (e.g., phycocyanin, phycoerythrin, astaxanthin, and etc.) (Chakdar et al., 2020).

Currently, most commercially obtainable recombinant technologies rely on host systems, which are organisms that can produce valuable proteins and bioactive compounds via genetic engineering, such as bacteria, yeast, transgenic animals, and transgenic plants. However, each host has their own benefits and drawbacks, depending on the target products. When eukaryotic plant compounds are the set goal, bacteria and yeast are not suitable because they lack post-transcriptional and post-translational mechanisms (e.g., glycosylation, splicing, and protein assembly) (Koo et al., 2013). Even though bacteria are frequently used for recombinant proteins, bacterial endotoxin and protease contaminants are concerned in biopharmaceutical products. Yeast is an excellent eukaryotic host because of its low cost and up-scalability, however, hypermannosylation, which commonly occurs in yeast, leads misfolded proteins and activity malfunction (Yusibov and Mamedov, 2010). Most biopharmaceutical products are manufactured in animal cells, but animal hosts still have some limitations, such as low yield, high cost, expensive medium, and virus contamination, making them unsustainable as a host in medical applications. Plant-based expression systems can solve the following problems, such as having a eukaryotic mechanisms, no hypermannosylation, and etc. However, plant hosts have to deal with some limitations and environmental issues, including the spread of genetically modified plants (GMO), allergic reactions to plant components, contamination of proteins, regulation of medical protein permission, and a long production period (Koo et al., 2013).

Eukaryotic algae, especially green microalgae, share evolutionary ancestry with land plants (Novoveska et al., 2019; Saini et al., 2019). They hold incredible metabolic potential and possess most criteria for being a good host of eukaryotic phytocompound expression. These criteria include: (i) microalgae are a various group of microscopic plants that share a common ancestor, thus it might have less complexity to modify their genetic pathway for producing plant chemicals, (ii) many microalgal species have ability to grow in extreme conditions, so the cost will be minimized related to no steady environmental conditions, (iii) post-translational modification pathways of microalgae are numerous to enable proper maturation for a variety of protein, especially for plant compounds (Scaife et al., 2015; Weiner et al., 2018).

Normally, microalgae are considered photoautotrophic organisms, whereas heterotrophic cultivation, which can use external carbon sources under dark conditions, has also been used to obtain high value products. Heterotrophs have many advantages compared to autotrophs, such as growing on a larger scale, having more FDA-approved standards and protocols for industrial fermenters, and ability to grow in higher cell density, among others (Rasala and Mayfield, 2015). Green microalgal hosts have been continually developed for expression. In this paper, several green microalgal hosts and their genetic toolboxes, including transformation methods, vectors, promoters, and selectable markers are presented, with a major focus on heterotrophic microalgae for phytochemical biosynthesis in an attempt to address the above concerns.

## Advantages of Heterotrophic Microalgae as a Host

**FIGURE 2:**
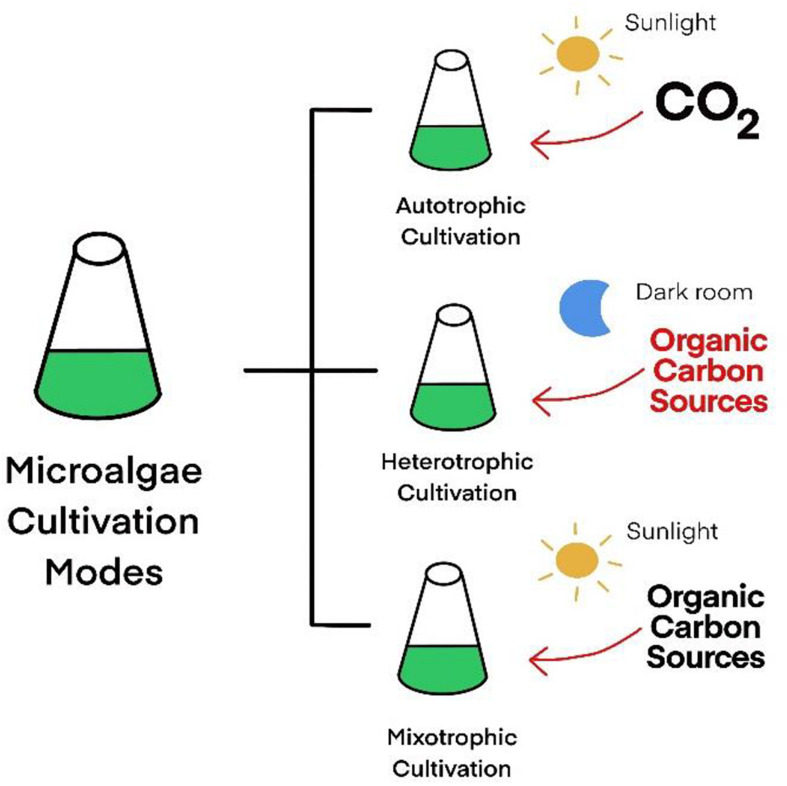
Microalgae cultivation status.

Microalgae are also known as single-cell algae that have a vital role in the food chain. Interestingly, microalgae can produce other nutrients that are also found in higher plants, including synthesizing lipids, fatty acids, proteins, nucleic acids, carbohydrates, fibers, starches, vitamins, and antioxidants (Klamczynska and Mooney, 2017). Unicellular microalgae present in a wide range of habitats and can be cultured in three cultivation conditions: autotrophic, heterotrophic, or mixotrophic mode (Figure 2). Autotrophic microalgae use energy from photosynthesis to grow, while some microalgae can grow in the dark using organic compounds as carbon and energy sources, which is called heterotrophic microalgae. Mixotrophic microalgae can use both supplied organic carbons and light energy in cultivation. Nowadays, many researchers have studied the production of pharmaceutical proteins, antibodies, and valuable compounds in microalgae (Koo et al., 2013; Dreesen et al., 2010).

Recently, attention has been drawn to microalgae as simple models for a sustainable source of high-value compounds, ranging from therapeutic proteins to biofuels (Rosenberg et al., 2008; Huang et al., 2010; Gong et al., 2011; Yang et al., 2016). Apparently, autotrophs and mixotrophs have drawbacks, which are described in detail below. Hence, the focus moves to heterotrophic microalgae that can grow well in the dark, like yeast and bacteria, by using simple carbon sources, such as glucose. Other advantages of heterotrophic microalgae for expression of phytochemicals include the following:

(1) Compared with traditional used host, prokaryotic hosts are the most commonly used platforms. Due to post-translational modification and protein localization are important for the production of phytocompounds or eukaryotic substances, whereas, prokaryotic *Escherichia coli* is not always the easiest hosts for this process (Yang et al., 2016). However another eukaryotic hosts including insect, mammalian cells, and transgenic animals may overcome these obstacles, but these systems might suffer from other limitations, such as virus contamination, proteolysis, expensive cost, incorrect glycosylation, high nutrient requirement, and long generation time (Gomes et al., 2016). Hence, alternative hosts are still needed. For example, eukaryotic microalgae, this is because they give the advantages of fast growing, low cost, ease manipulation, and etc. (Yang et al., 2016). Moreover, they allow glycosylated proteins to be secreted into the cell from post-translational modification pathways (Lauersen et al., 2013). The comparison of advantages and disadvantages to produce plant compounds among host systems and other methods is summarized in Table 1.

**TABLE 1:** Brief comparison of merits and demerits among different host systems and plant cultivation.

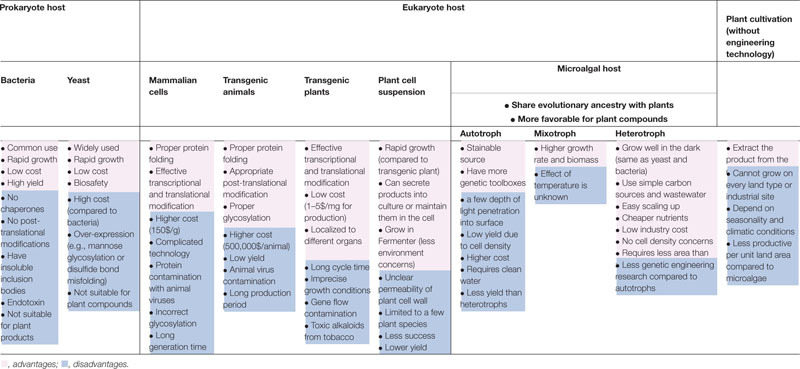

**FIGURE 3:**
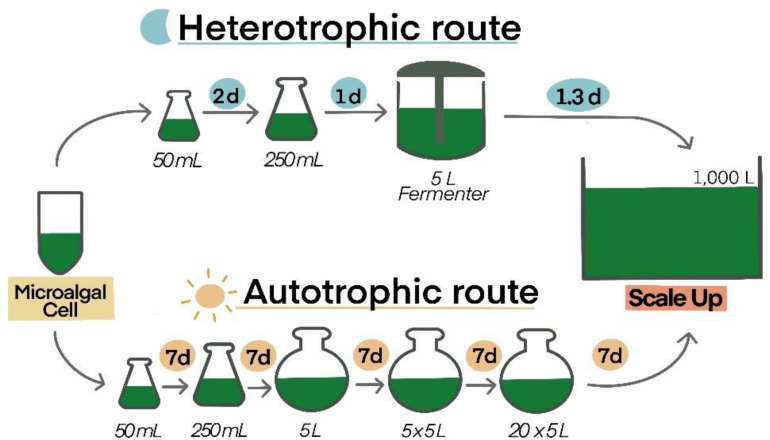
Comparison of time consumption between autotrophic and heterotrophic routes of *Chlorella vulgaris*. Culture volumes (liters) and duration (d: days) of each scale up step are demonstrated (Barros et al., 2019).

(2) Compared to plant cultivation and synthesized phytochemicals, microalgae are easily scalable in fermenters or bioreactors compared to plant cultivation because they can be constructed on any land type or industrial site (Melis, 2012). This shows that microalgae are non-seasonal, not dependent on climatic conditions, and do not need arable land (Lopes et al., 2019). Even if plant compounds can be synthesized by using chemicals instead of cultivation, in some cases, the complexity of their structure, which requires difficult multistep reactions, leads to high costs, very low yield, and unwanted effects for pharmaceutical product. Synthesized compounds are designed and utilized synthetic DNA parts, whereas metabolic engineering involves protein and pathway optimization for improving the yield of products (Stephanopoulos, 2012).

(3) Compared to transgenic plant, microalgae share evolutionary ancestry with land plants. That means genetic manipulation techniques might be easily adapted to microalgae, such as codon optimization, intron addition, expression methods, and vectors (Scaife et al., 2015). For transgenic plants to express any gene, there are limitations. First, plant cell suspension culture or plant tissues can grow in fermenters, but they are limited to a few plant species compared to a wide range of microalgae. Microalgae cells might be more favorable for plant compounds production than yeast, bacteria, or others hosts because microalgal cellular environments are suitable for those exogenous plant enzymes. Additionally, microalgae metabolism contains production of precursors which are more associated with phytocompounds production more than prokaryotic host (Lauersen, 2018). For transgenic plants, there are only a few examples that have been commercially developed and there are still bottlenecks for commercial production, compared to a microalgal host. Second, the procedures to transform genes take longer periods of time than in a microalgal host; for example, expression in tomato requires more than a year, while green microalgae need a few days (Canto, 2016). Moreover, microalgae require only a few months to scale up compared to transgenic plants; for instance, tobacco plants take 6 months to grow after regeneration. However, apart from Faè et al. (2017) research, it is assumed that the specific activity of the enzyme produced by *Chlamydomonas* and tobacco are alike, as both proteins synthesis machinery in chloroplast is highly conserved. Faè et al. (2017) suggested that algal molecule farming is still desirable for high value pharmaceutical production. Third, there are concerns about transgenic plants transferring genes to the environment via pollen, which might not occur in microalgae, especially in heterotrophic microalgal hosts because there is no in and out for contaminated sources in the fermenter. Forth, product expression from plants might be contaminated with agrochemicals and fertilizers, so downstream cultivation after expression should be considered (Gomes et al., 2016). Finally, the main differences between the application of higher plant systems and microalgae for biotechnology is the scalability of cultivation in fermenters (Yu et al., 2013).

(4) Compared among microalgae cultivation, heterotrophic microalgae have more benefits, such as cheaper nutrients, low cost of instruments, and easy to operate and maintain. They can be adapted to a large scale with no cell density and less-stress concerns in only a few weeks (Yang et al., 2016). Autotrophs use CO_2_ and light as inorganic carbon and energy sources, whereas heterotrophs use organic carbon as a source of carbon and energy (Lopes et al., 2019). Several species including *Chlamydomonas reinhardtii*, *Auxenochlorella protothecoides*, *Chlorella pyrenoidosa*, *C. vulgaris*, and *C. zofingiensis* can be grown in low-cost industrial waste products (Abreu et al., 2012). Although autotrophic microalgae can be cultured in large scale production, there are some disadvantages: only a few centimeters of light/sunlight penetrate the surface, which reduces cell growth; high cell density is related to low yield; high cost of transparent material for gaining light; difficult to design narrow photo-bioreactors; significant financial investment for energy use and maintenance; difficult to maintain in mono-culture; need continuous and clean water; and not compatible with pharmaceutical or food production (Wolf et al., 2016; Barros et al., 2019). For biomass yields, heterotrophs make 50–100 g/L of cell dry weight. This number is higher than that of autotrophs, which reach a maximum 30 g/L of cell dry weight (Perez-Garcia et al., 2011). Moreover, under heterotrophic conditions, *Chlorella* growth is approximately 5.5 times higher than cultures under light conditions (Yu et al., 2013). In particular, the period for scale-up of heterotrophic microalgae is shorter than autotrophic microalgae (Figure 3). In addition, the overall area cultivation for heterotrophs is 12 times less than that of autotrophs (Barros et al., 2019). From one study, it was shown that there is high impact of heat and energy use for autotrophs, but for heterotrophic microalgae, these are controlled by glucose feedings (Smetana et al., 2017). The carbon intermediates of heterotrophs are transformed into main metabolic pathways, replacing photosynthetically produced molecules (Morales-Sánchez et al., 2015). While, some autotrophs are able to grow in the dark, the central carbon metabolism of autotrophic growth involves incomplete pathways or the absence of an enzymatic reaction, which is a primary cause of obligation to consume vital substrates, particularly sugars, and other carbon sources (Morales-Sánchez et al., 2015). Thus, culturing heterotrophs in a fermenter might be a better option.

(5) In medicine, where production for humans is regulated under strict safety aspects (Gellissen, 2005), there are a variety of suitable microalgae that can be selected from their Generally Recognized as Safe (GRAS) status, depending on the purpose. For example, *Chlorella vulgaris* (a green alga) is normally used as a food additive, feed for animals, and diet supplements. Moreover, *Arthrospira platensis* (*Spirulina platensis*; a cyanobacterium), which has high protein and nutrient contents, is consumed as food and feed (Yaakob et al., 2014). Therefore, this is a great opportunity to develop these microalgae as a host.

(6) When considering the environmental impact of host systems, there are three main indicators, namely less greenhouse gas emissions, low water supply, and efficiency of land use. Heterotrophic microalgae offer these three main criteria. A study found that whole algae protein has a lower water footprint than beef and whey but more protein per hectare than other sources (Klamczynska and Mooney, 2017). Moreover, using simple media for the cultivation of algae is as low as $0.002 per liter compared to mainly using mammalian hosts, which cost $150 per gram (Taunt et al., 2018).

A suitable heterotrophic microalgae should have the following essential criteria: ability to grow without light, can be cultured on inexpensive and easily sterilized media, rapidly adapt to new surroundings, and the ability to endure hydrodynamic stress in fermenters and other equipments (Chen and Johns, 1996; Wen and Chen, 2003). Many factors have to be considered for culturing heterotrophic microalgae, including temperature, medium salinity (NaCl), pH, and dissolved O_2_. In the heterotrophic status of *Chlorella sorokiniana*, high aeration increased cell growth, fatty acid yield, and unsaturated dienoic and trienoic fatty acids; conversely, this decreased cell lipid content (Chen and Johns, 1991). In heterotrophic metabolism, carbon is broken down in the same way used by bacteria. Complex molecules, like starch, are metabolized via the Embden-Mayerhoff-Parnas Pathway (EMP pathway or glycolysis) or the Pentose Phosphate pathway (PPP). However, heterotrophic culturing has some limitations, including high cost by adding more organic substrates, contamination or competition with other microorganisms, and unproduced light-induced metabolites (Perez-Garcia et al., 2011).

## Genetic Engineering of Microalgae

Recently, the development of microalgae biotechnological platforms has been continually progressed, especially from a genetic engineering perspective. Microalgae have potential to act like a cell factory to produce other compounds and proteins at economical levels. To date, over 40 different microalgae species, such as *Chlamydomonas reinhardtii*, *Dunaliella salina*, *Chlorella vulgaris*, and *Haematococcus pluvialis*, have been successfully genetically manipulated. The available genetic tools are for both nuclear and chloroplast transformation for *C. reinhardtii* and *Phaeodactylum tricornutum*, however, there is still a lack of genetic toolboxes and applications compared to others host systems. In the green microalgal host area, algal genome data and transformation protocols are available (Gangl et al., 2015). However, research is rarely found for heterotrophic microalgal hosts, even if they are normally used on an industrial scale. These hosts have less limitation in the recombinant technological field compared to autotrophic microalgae and some other hosts.

Microalgae generally consist of nuclear, mitochondrial, and plastid genomes (Radakovits et al., 2012). Compared between nuclear and chloroplast transformation, which are crucially different (Table 2), chloroplast transformation allows higher accumulation of the desired protein (Faè et al., 2017). Several complete genomic resources are available in some species, such as the model of green microalga *C. reinhardtii*. Recently, many reports described genetic engineering of the chloroplast, which has small genome (205 kb) and less-complexity with only 99 genes. Although chloroplast transformation is also feasible for plenty of plant species, such as tobacco, tomato, and petunia, there are still fundamental challenges and less achievement reports than those for nuclear transformation (Gong et al., 2011). Nowadays, low cost sequencing technologies make more fully sequenced genomes of algae strains available (Jaeger et al., 2017). However, engineering strategies across all microalgae are difficult because their genetic contexts are highly specific, variable, and often poorly understood. Even if *C. reinhardtii* was a model organism, there is lack of a viable commercial production process and food safety (Taunt et al., 2018).

**TABLE 2:** Differences between nucleus and chloroplast transformation; adapted from Rasala and Mayfield (2015).

**Genome engineering**	**Nucleus**	**Chloroplast**
Gene expression mechanism	Eukaryotic	Prokaryotic
Silencing	More	Less
Protein localization	Cytoplasm, nucleus, chloroplast, ER*, mitochondria, secretion	Chloroplast
Modifications	Phosphorylation, glycosylation, disulfide bond	Phosphorylation, disulfide bond
Accumulation levels	Low (as high as 0.25% TSP reported)	High (1-21% TSP*)
Transformation methods	Electroporation, particle bombardment, glass beads, PEG*, Agrobacterium	Particle bombardment, glass beads, Agrobacterium
Integration mode	Non-homologous end joining	Homologous recombination
Inducible gene expression	Nutrient, chemical, physiological	Light inducible

*ER, endoplasmic reticulum; TSP, total soluble protein; PEG, polyethylene glycol mediated transformation.

The chloroplast of green algae consists of gene machinery, including the ribosomes and translation factors, however, it is not similar to bacteria because the chloroplast contains a wide range of chaperones, protein disulfide isomerase, and peptidylprolyl isomerases. These chaperones aid in complex protein folding, and as a consequence, this unique biochemical environment allows for the expression of high-valuable biopharmaceuticals (Rasala and Mayfield, 2015). In fact, heterotrophic processes might limit the development of chlorophyll because it is no longer needed for metabolism (Klamczynska and Mooney, 2017).

Currently, several new expression systems are commercially available, but some of them are private and need licensing. Many researchers are looking for other microalgal hosts because of the advantages of rapid growth, low cost, cheap medium, ease of culture, and board industrial applications. For example, *Chlorella* which has been chosen because for its fast growth with high cell density under various culture modes and adaptability to different conditions is interesting as a potential newcomer host for heterologous protein expression (Yang et al., 2016; Klamczynska and Mooney, 2017). They can be cultured in both autotrophic and heterotrophic culture.

Moreover, reducing culture time and high biomass might be better options for choosing microalgae that can double their biomass in less than 24 h, such as *Chlorella sorokiniana*, which has a doubling time of less than 3 h (Sorokin, 1967) and a new transgenic time of around 2 months on an industrial scale (Mayfield et al., 2007). One of the fastest growing species is *Chlorella vulgaris*, thus this specie is another promising algae model for genetic engineering. *Chlorella* species are future hosts for protein and glycoproteins, while diatom *Phareodactylum tricornumtum* has been shown to produce a fully functional anti-hepatitis antibody with high-mannose glycan (Mathieu-Rivet et al., 2014; Yang et al., 2016; Vanier et al., 2017).

For human consumption, *Spirulina* and *Chlorella* are best-known for nutritional properties. They are consumed in many forms, such as tablets, capsules, and liquids (Aron et al., 2020; Khoo et al., 2020), so this familiarization might be the answer for producing recombinant biopharmaceuticals in these microalgal hosts. Although there are many reports of successful recombinant technology in algae, there is only one report of transferring recombinant production to a large scale (Gangl et al., 2015). This shows that there are still gaps in the knowledge transfer from a lab scale to industrially relevant growth conditions for recombinant production. However, the cheap cost of culturing, potential for large-scale in fermenter growth, and many GRAS status species are advantages of heterotrophic microalgae. In the future, gaps might be filled in as the industry is continually growing.

## Genetic Transformation of Microalgae

There are many transformation methods for the delivery of genes into algal cells, including agitation by glass beads or silicon carbide whiskers, electroporation, polyethylene glycol (PEG) mediated transformation, particle bombardment, and Agrobacterium-mediated transformation (Kim et al., 2014). The cell wall of algae is a physical barrier for foreign DNA because of the cell membrane. Hence, many transformation methods depend on cell excluding the cell wall, which is called protoplasts. For instance, *Chlamydomonas* cell walls, which consist of glycoproteins and cellulose or chitin, can be degraded by autolysins, while *Chlorella* cell walls are composed of sugar polymers that can be degraded by sugar digesting enzymes (Kim et al., 2014). The most frequently used methods are particle bombardment and electroporation, however, agitation methods that have a lower transformation rate are often used because of the minimal equipment required. In contrast, Agrobacterium-mediated transformation has not been extensively used, and less information is known about its use in microalgae (Barrera and Mayfield, 2013). This transformation method is normally used in plant systems, thus, some researchers adapted this method for microalgae. From one report, some microalgae were electro-transformed, but the transformants were just a few. While Agrobacterium-mediated transformation had much more transformation rate when compared between ten microalgae (Suttangkakul et al., 2019). Thus, choosing the transformation method is determined by the cell size, nature of the cell wall, species, target organelles, cost, and especially the aim of the interested product. A comparison and some limitations of transformation methods are shown in Table 3.

**TABLE 3:** Comparison between transformation methods.

**Methods**	**Techniques**	**Cost**	*Trans*- formant*	**Limitations**	**References**
Glass bead	DNA delivery is based on agitating protoplasts or cell wall-deficient using glass beads or silicon carbide whiskers with foreign DNA.	Low	1,000	- Effect of shear stress - Requires cell wall-deficient strain	Kim et al., 2014

Particle bombardment	-DNA-coated gold or tungsten micro-particle is delivered by using a specialized tool. -Does not require removal of the cell wall.	Very high	Very good	- Expensive tools - Size of the particle is an important factor for nuclear or plastid transformation (smaller size increases penetration) - Low repeatability - Complex operation process	Potvin and Zhang, 2010; Kim et al., 2014

Agro bacterium	Using Agrobacterium, DNA is transformed into host cells.	Low	20x glass bead	- Related to biological compatibility - Less known in microalgal host	Barrera and Mayfield, 2013

Electroporation	Using an electric pulse to push DNA into cells	High	2,500–7,137	- Uses specialized equipment - Requires strains without or a reduced cell wall - Random integration of genes - Optimal conditions depend on species (osmolality, temperature, concentration of DNA, voltage, electroporation buffer, pulse length, field strength, and capacitance) - If extreme conditions are used, it may cause a low cell viability due to the presence of cell walls.	Barrera and Mayfield, 2013

PEG-mediated	DNA delivery is based on agitating protoplasts or cell wall-deficient with PEG and foreign DNA.	Medium	356–2,250	- Requires cell wall-deficient strain - Factors affect the transformation (starting material, Agrobacterium density, co-cultivation conditions, acetosyringone concentration, etc.)	Cha et al., 2012

*transformant unit: cfu per μg DNA.

### Vector Construction

Common strategies have been considered, including increasing transcription levels by choosing strong promoters with appropriate enhancers and leader sequences, the improvement of translation via codon usage optimization, control of transgene copy number, gene product targeting by using signal peptide, and host genome position (Table 4).

**TABLE 4:** Some microalgal expression methods, vectors, and selectable markers.

**Strains**	**Plasmids**	**Promoters**	**Expression methods**	**Selectable markers/Reporter genes**	**References**
*Scenedesmus acutus*	pCXSN-GEP	psaD, RBCS2	Agrobacterium	Hygromycin B	Suttangkakul et al., 2019
*Chlamydomonas reinhardtii*	pET-vp28	atpA	Glass bead	Spectinomycin	Kiataramgul et al., 2020
	pER123	–	Glass bead	Paromonycin	Mooi et al., 2018
	pSL18_HR	HSP70A	Electroporation	Paromomycin	Perozeni et al., 2018
	Atp B-int	psaA	Helium gun bombardment	Spectinomycin	Faè et al., 2017
	pChlamy3	LIP	Glass beads	Hygromycin	Baek et al., 2016
	pMS4-3	B12-responsive element	Electroporation	*METE* reporter gene	Helliwell et al., 2014
	pCRD1-5	CYC6	Electroporation	Luciferase	Quinn et al., 2003
	cabII-1 chimeric	CABII-1	Electroporation	GUS	Blankenship and Kindle, 1992
*Phaeodactylum tricornutum*	pHY21	Pt211	Electroporation	GUS, DGAT2	Zou et al., 2018
	pHY11	FCP	Electroporation	Chloramphenicol acetyltransferase (CAT)	Xue et al., 2015
*Chromochloris zofingiensis*	pCZT1	RBCS	Gold bombardment, electroporation	PDS gene for herbicides	Mooi et al., 2018
*Chlorella pyrenoidosa*	pGreeII 0029	Ubiquitin	Electroporation	NptII, eGFP	Run et al., 2016
*Chlorella vulgaris*	pCAMBIA1304	CaMV 35S	Electroporation	Hygromycin	Koo et al., 2013
	pPt-ApCAT	NR gene	Electroporation	Chloramphenicol	Niu et al., 2011
*Chlorella ellipsoidea*	pSoup	NIT1	Electroporation	NptII	Bai et al., 2013
*Claculinopsis fusiformis*	*p*ble	Pδ	Bombardment	Zeocin	Fischer et al., 1999
*Dunaliella salina*	pUCG-Bar	GAPDH	Electroporation	Herbicide PPT	Jia et al., 2012

To generate a plasmid vector, which is the critical step for genetic transformation, the vector might include the genetic elements (e.g., promoters, enhancers, reporters, marker genes, and codon usage). Promoters are a crucial factor for gene expression and have a significant transcriptional regulation effect. There are different types of optional promoters. In general, high gene expression is positively correlated with a strong promoter. Some native promoters, including heat shock protein 70A (HSP70A), Rubisco small submit (RBCS2), or photosystem I protein D (psaD), are used in *C. reinhardtii* (Kim et al., 2018). Moreover, an inducible promoter is the one feasible choice for solving the effect of some proteins that might work on the growth of transgenic cells. Interestingly, some heterologous promoters that are widely used in plant transformation have been utilized in microalgae, such as the cauliflower mosaic virus (CaMV) 35S promoter and p1’2’ *Agrobacterium* promoter, which drives the expression of GUS reporter genes (Jaeger et al., 2017). Thus, this can be a good sign for using microalgae as a plant compound host. Additionally, other commonly used promoters for microalgae are RBCS2, psaD, fcp, Pδ, GAPDH, CABII-1, NIT1, Ubi1-Ω, LIP, B12-responsive element, Actin1, NR gene, and CYC6 promoters. Currently, some researchers suggested that synthetic algal promoters (saps) can be used based on the characteristics of strong promoter motifs (Scranton et al., 2016). According to the research on *Chlorella* sp., expression promoters are in the early stages of development; only heterologous promoters from plant systems were used, such as 35S, ubiquitin, and NOs promoters (Run et al., 2016). Thus, further studies on expression and gene regulation in these microorganisms are necessary. From some studies, it was suggested that even when using the same construct, there are still variable expression patterns among different transformants, related to the number and location of recombination events. With supporting enhancers, transgene expression can be activated, no matter where the location of a target promoter is (Smallwood and Ren, 2013).

### Reporter Genes

Reporter genes that encode easily recognizable proteins are useful for studying transformation efficiency, protein localization, and stability of transgenes. While selectable markers are proteins for helping the selection of positive transformants by being resistant to antibiotics (e.g., spectinomycin, kanamycin, erythromycin, chloramphenicol), herbicides (e.g., sulfometuron methyl, glufosinate, norflurazon), or having a function as a metabolic mutant (e.g., photoautotrophic growth, arginine free media, nitrate salt presented media) (Morales-Sánchez et al., 2015). Although antibiotic resistance genes are usually used for selecting the transformant, metabolic selection is considered to be environmentally friendly (Doron et al., 2016). Particularly, stable transformation depends on the use of a suitable selection marker.

### Condon Optimization

Codon optimization is also important to consider because it significantly affects translation efficiency and protein expression levels. Codon bias from tRNA abundance can be quite different not only for various species genomes but for various organelles. The length of vector construction can lead to false positive transformants in microalgal hosts. The efficiency of positive transformants can range from 2–50% depending on the construct (Baier et al., 2018). Microalgae are still being used more than *P. tricoronutum* (diatom) because diatom is sensitivity and slow growth, even though they have less-complex genetic data.

When DNA synthesis is more reliable and cheap, it may soon be possible to design and construct complex metabolic pathways in microalgae (Lauersen et al., 2018). In recent years, many vectors, toolboxes, and strategies have been developed for the model microalgae *Chlamydomonas*, but these cannot be applied for all microalgae. Until now, non-model microalgae were still a challenge because of the lack of development in tools and strategies (Suttangkakul et al., 2019). In some cases, they can produce recombinant proteins in the same way as *Chladmydomonas reinhardtii*.

### Protein Degradation

Proteases can degrade foreign proteins, so knockdown technologies, such as RNAi, are used to limit proteolysis. Methods to control this limitation are still required for further improvement in microalgae. Furthermore, foreign protein toxicity should also be considered; for example, the cholera toxin-B subunit is toxic to tobacco cells only when expressed in the cytosol (Daniell et al., 2001). Thus, similar aspects should be considered when using microalgae as a host.

### Secretion Product

In eukaryotes, secretion can ensure proper glycosylation of proteins, which plays an important role in determining yield, biological function, stability, and half-life of production. Nevertheless, these mechanisms of protein glycosylation in higher plants remain unknown (Mathieu-Rivet et al., 2014). Therefore, secretion of expressed protein into the medium is widely used in heterotrophic microalgal hosts (Demain and Vaishnav, 2009). In general, secretion yields more than 10 mg/L are a minimum for commercial processes (Hellwig et al., 2014), while heterotrophic microalgae could have a yield more than 1 g/L. In 2017, reports supported potential of transgenic microalgae as a host for the secretion of recombinant production (Ramos-Martinez et al., 2017).

## Microalgal Engineering for Phytochemicals Production

Microalgae have great potential to produce novel metabolites and other high-value compounds. Plant secondary products or specialized metabolites are some of the most crucial target compounds (Gangl et al., 2015). These plant compounds have been used in many areas, including pharmaceuticals, chemicals, food industries, and medicines. Moreover, approximately 50% of all approved medicines are from plant compounds (Lassen et al., 2014). Recently, some researchers and biotechnologies aim to replace many types of plant compounds with microorganisms via genetic technology because various substances are normally found in small amounts in plant, which means that some parts of the plant are wasted biomass. Moreover, there remains an imperfect production of the chemical on an industrial scale for some types of compounds. More recently, microalgae have become fascinating and interesting hosts to produce heterologous isoprenoids, which are high-value plant secondary metabolites. Researchers have strongly suggested that pharmaceutical products, such as terpenoids, are not only produced in plant chloroplasts but also in microalgal chloroplasts (Bock and Warzecha, 2010). Some algae accumulate a large percentage of triacylglycerol (TAGs), which is similar to those found in plant oils (Hu et al., 2008). Unfortunately, some high-value compounds, such as terpenoids, are less expressed in *E. coli* and *Saccharomyces cerevisiae* because those compounds need special localization and post-translational modification (Chemler and Koffas, 2008). In 2018, the invention of producing cannabinoids, which is a phytocompound, in an algae host was presented for a patent. The expression systems and method can convert a fatty acid into a cannabinoid in an algae host (Laban, 2019). Recent studies have shown the ability of microalgal host to express, post-translationally modify, fold, and secrete plant chemicals and proteins (Table 5).

**TABLE 5:** Recent phytochemicals manufactured in microalgae.

**Microalgal hosts**	**Phytochemical productions**	**Functions**	**Cultivation modes**	**References**
***Porphyridium* sp.**	***• Carbohydrates*:** Exopolysaccharides (EPS) *•* **PUFAs:** Arachidonic acid (AA) *•* ***Protein-pigment complexs*:** B-phycoerythrin, etc.	High-value bioactive substances (food, medicine, nutrition)	Phototroph, Mixotroph, Heterotroph	Li et al., 2020
***Chlamydomonas reinhardtii, Synechococcus elongatus***	***• Cannabinoids*:** delta-9-tetrahydrocannabinoid (Δ9-THC), cannabidiol (CBD), etc.	Treat a wide range of medical conditions (e.g., AIDS, neuropathic pain, spasticity)	Phototroph	Laban, 2019
***C. reinhardtii***	*•* ***Hydrocarbons*:** terpenoids	High-value plant secondary metabolites (antioxidant, dietary, supplement, pigment)	Phototroph	Lauersen, 2018
	*•* ***Metabolites*:** Cytochrome P450 enzymes (P450s) which is involved in the biosynthesis of complex plant metabolites (e.g., paclitaxel accumulation in plant; *Taxus baccata*)	Paclitaxel as a natural source cancer drug	Phototroph	Gangl et al., 2015
***Scenedesmus* sp.**	*•* ***Pigments*:** β-carotene (red-orange found plants and fruits), Lutein	Health food, dietary, supplements, cosmetics, feed	Phototroph	Chen et al., 2017
***Dunaliella* sp.**	***• Pigments***: β-carotene, astaxanthin	Food coloring, antioxidant, anti-allergic, anti-inflammatory	Phototroph	Saha et al., 2018; Barkia et al., 2019
***Haematococcus* sp.**	*•* ***Pigments*:** β-carotene, astaxanthin	Antioxidant, anti-inflammatory		Barkia et al., 2019
***Chlorella* sp.**	*•* ***Pigments*:** lutein (a large amount of lutein present in marigold flowers) • ***Proteins*:** whole, dried microalgae	Antioxidant, dietary, cosmetic, pigment	Phototroph, Heterotroph	Sun et al., 2016
***C. pyrenoidosa***	• ***Micronutrients*:** polyphenols (present in diverse plants)	Pharmacological activities, antioxidant	Phototroph	Olasehinde et al., 2017
***Neochloris oleoabundans***	• ***Fatty acids*:** triacylglycerols (TAGs) (major component of vegetative oils)	Great nutritional, nutraceutical value, edible oils, and industrial purposes.	Phototroph	Chungjatupornchai et al., 2019
***Botryococcus braunii***	• ***Hydrocarbons*:** alkadiene, botryococcene • ***Metabolites*:** phenolics, carotenoids	high-quality fuel applications, antioxidant, medical values	Phototroph	Cheng et al., 2018; Kempinski and Chappell, 2019
**Green algae, *Volvox carteri***	• ***Phytohormones*:** auxin, abscisic acid, cytokinin, ethylene	Plant hormone	Phototroph	Lu and Xu, 2015

Additionally, some studies have attempted to convert autotrophic algae into heterotrophs by using genetic manipulation to adapt microalgae to different growth conditions (Taunt et al., 2018). However, some studies have reported that the yield of *Chlorella* was 200 ng/L to 11.42 mg/L, which is lower than other hosts, including plants (0.1 μg/L to 247 mg/L), mammalian cells (0. 55–80 mg/L), and insect cells (80–300 mg/L). Fortunately, rapid growth of *Chlorella* might gain higher yield (Yang et al., 2016).

## Challenges of Microalgal Hosts

The major challenge is bacterial contamination in heterotrophic microalgal culture and biomass since the faster grow of bacterial populations is a consequence of commercial applications. Thus, sterilization steps are necessary, which cause a higher cost on a large scale due to equipment demands, such as autoclaves, laminar flow cabinets, and boilers. Besides, the use of industrial wastes in the culture medium could be risky for high microbial load. However, lower cost sterilization methods, including sodium hypochlorite usage, are another option to investigate for replacing expensive sterile tools on a large scale (Peiris et al., 2012).

Another major concern is the need for aeration and efficient mixing in the liquid medium for avoiding transfer limitations that can reduce cell biomass and yield (Lopes et al., 2019). In this sense, technological development of bioreactors is required to provide adequate oxygen under gentle stirring at a large scale without the presence of dead zones. Today, the limitations of industrial scale rely on the future development of a bioreactor which can operate in a larger scale (Severo et al., 2019).

Additionally, microalgal hosts, especially under heterotrophic cultivation, are still challenged by some obstacles for phytochemical production. Microalgae recombinant techniques for molecular development, including enhancing transcription, improving translation efficiency, and minimizing post-translational degradation, and process development, such as improving cultivation methods and optimizing scale-up culture, are needed. In the United States and Europe, biopharmaceutical industries are using microbial fermentation and mammalian cells for production. Host system research using microalgae should be encouraged over other hosts. Although, genetically modified microalgae have less of a chance to survive in the environment, it is suggested to analyze the risks before staring industrial production outdoors (Wijffels et al., 2013).

## Key Market Trends

**FIGURE 4:**
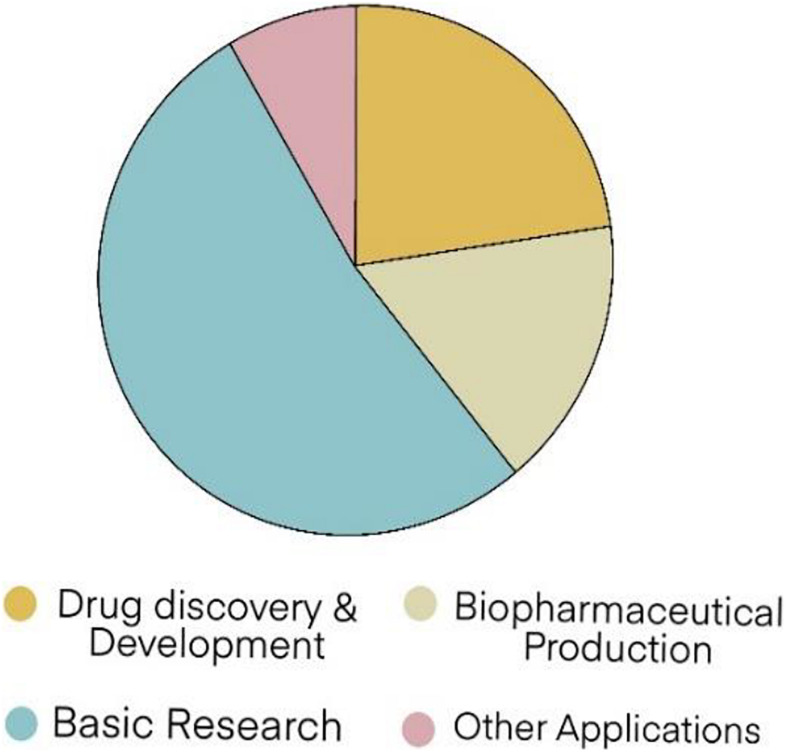
Recombinant protein global market the forecast trends in 2025 (Coherent Market Insights, 2020).

The growth of valuable protein and compound markets has continually increased in research and development. Therapeutic applications from biopharmaceuticals have become bestsellers for the treatment of many chronic conditions, like diabetes, cancer, psoriasis, multiple sclerosis, rheumatic diseases, and inflammatory bowel diseases. The biopharmaceutical market was valued at approximately US$ 199.7 billion in 2013 and might reach US$ 497.9 billion in 2020; hence, an overall compound growth rate of 13.5% per year (Xu and Zhang, 2014). Many application trends of recombinant proteins in the global market in 2025 are shown in Figure 4 (Coherent Market Insights, 2020).

Over 50 different biopharmaceuticals have been successfully produced in microalgae (Lauersen et al., 2013). Microalgae represent a third-generation biofuel and an energy source. Moreover, their short life cycle, environmental adaptation, and wide range of distribution serve as good criteria for economic systems. According to global market research, various products from algae are expected to grow at 4.2% annual growth rate from 2018 to 2025. Furthermore, a total market value is more than 3.4 billion US$, while no biopharmaceuticals produced from microalgae have been approved for commercial production (Taunt et al., 2018; Kumar et al., 2020). However, for reasons of high cost and unavailability of genetic information for commercially suitable strains, they have not yet reached industrial maturity and commercial success. So far, a considerable effort has been given to tackle the bottleneck of various methods, including various nutritional-, environmental-, and physiological alteration of cultivation, metabolic and genetic engineering (Pierobon et al., 2018; Chen and Lee, 2019). To meet large market demand, a high technological level and the use of mechanized harvests are required. Exploring the integration of new efficient technology of downstream processes including extraction, concentration, conversion, and purification of recombinant product from microalgae should be considered in future studies.

**FIGURE 5:**
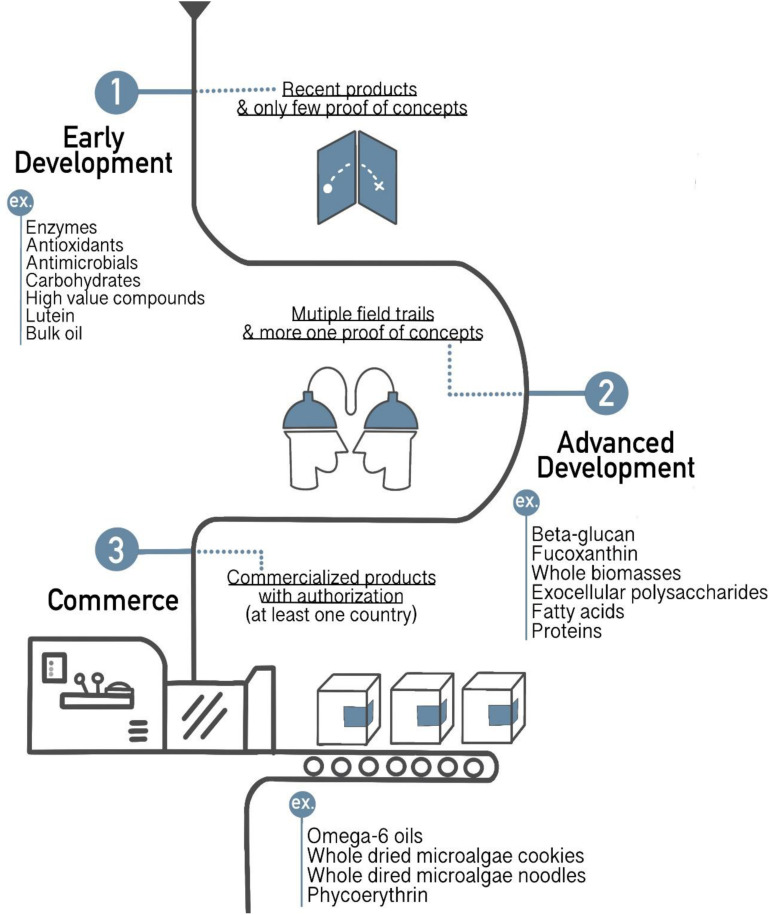
Several new products derived from microalgae in different stages (early development, advanced development, and commerce) of development (adapted from Jacob-Lopes et al., 2019).

To date, economic feasibility of some heterologous production will not be achieved with microalgal host, for example, sesquiterpenoid cosmetic and perfume have already been produced by microbial fermentation in the market under the name Clearwood by Firmenich (Lauersen, 2018). However, other productions still have been possibly produced in microalgal host, the return on investment can be achieved in short term. Recently, microalgae productions are continuingly developed in three stages of microalgae-based process developments (Figure 5). Commercialized microalgae products are sold on the market with authorization at least one county, such as omega-6 oils, whole dried microalgae cookies, whole dried microalgae noodles, and phycoerythrin. Some of the products are in advanced development which is in the multiple field trails and has more than one proof of concepts, including beta-glucan, fucoxanthin, whole biomasses, exocellular polysaccharides, fatty acids, and proteins. However, most of them are in early development stage that has only few proofs of concepts namely enzymes, antioxidants, antimicrobials, carbohydrates, lutein, bulk oil, and high-value compounds. Furthermore, demanding of high value compounds is increasing. For instant, the high value pigments like β-carotene make a selling price up to US$ 790 per kg (Jacob-Lopes et al., 2019). Recently, the carotenoid market has been reached US$ 1.53 billion until 2021 (Fernandes et al., 2018). Especially, heterotrophic microalgae have much attention for commercial applications because they overcome the difficulties of supplying CO_2_ and light compared to autotrophic microalgae (Hu et al., 2017). The cost of dry biomass for heterotrophic cultivation was US$ 2, whereas autotrophic cultivation was around US$ 11. Nevertheless, through the economic aspect, the main costs of heterotrophic cultivation are the set-up, equipment costs, and organic carbon source costs (Lowrey et al., 2015). About 80% of production costs spend to culture medium, so the replacement of alternative organic carbon sources can reduce approximately 40% (Santos et al., 2017). While many species of microalgae can be cultured in wastewater to reduce the costs of carbon source and other nutrition, they can use organic carbon and inorganic N and P from wastewater and also remove heavy metals (Jareonsin et al., 2019). Therefore, researchers are more likely to use wastewater from industrial applications, including livestock, kitchen, or pig wastewater on heterotrophic microalgae to enhance the economic feasibility and sustainability of production (Qin et al., 2019). However, the production of biopharmaceutical products might be challenged by using those wastewaters because of safety concerns.

Owing to biosafety concerns, the way to the world market requires approval of all genetically modified organisms (GMOs). Some organizations, such as European Food Safety Authority (EFSA)^1^ and OECD meeting on the Biosafety and Environmental Uses of Micro-Organisms, prepared a guidance protocol for risk assessment of genetically modified microorganisms (OECD, 2015). For instance, the protocols recommended that GM microorganisms should be grown in closed bioreactors, tubular reactors, or polyethylene sleeves, additionally, selection markers should be removed. Once the genetically improved strain is developed, biosafety will define its commercial success.

## Conclusion and Future Views

Tremendous breakthroughs in the new discovery of novel expression platforms for producing biopharmaceuticals or phytochemicals are needed. Heterotrophic microalgae are a sustainable and scalable host for recombinant technology. Microalgae share many attributes with higher plants, such as glycosylation patterns and having low risk of contamination by viruses or prions. Unlike higher plants, the closed-system of heterotrophs in fermenters is attractive because of safety aspects for biopharmaceutical products, cost-effectiveness, well-controlled environment, fast growth, and high yield on a large scale, suggesting the use of these organisms as alternative biotechnology. Thus, the genetic tools and design concepts of heterotrophic microalgae should be developed for increasing the number of known microalgae species under heterotrophic conditions.

Microalgae cultivation is well known to be the most profitable business in biotechnological industry since it has less waste. Additionally, the development of other GRAS species that have been grown commercially, such as *Chlorella* sp., *Dunaliella salina*, and *Haematococcus pluvialis*, may provide opportunities for reducing costs and scaling-up; moreover, these promising hosts will help to expand the various applications for recombinant microalgae-based production. Apparently, expanding basic or applied research for the use of autotrophic and heterotrophic microalgae is necessary.

The challenges to meet the economic demand are multifaceted, including quantities, qualities, and cost-effectiveness. Improving yield and product quality in some microalgal hosts remain to be addressed. A small number of microalgal hosts are approaching commercialization as the demand for therapeutics and other production is continually growing. These still remain some limitations for being microalgal host, such as difficult engineering due to the lack of a high-efficiency genetic toolbox (especially for heterotrophic microalgae), less-available molecular specific toolkits, short-term stability genetic system, and less efficient manipulation outside laboratory. To counter these limitations of phytocompounds using microalgal host, the basal study of molecular elements, such as identification and cloning of promoters, enhancers, and terminator should be studied up more. The innovation and toolkits for microalgae are also need to be specifically improved. Using industrial or agricultural waste contained with less microbial load should be adapted to medium for sustainability and saving cost for industrial scale. Indeed, fundamental knowledge and research are also necessary, making more research on various cultivation conditions a good option within the next few years.

Many plant chemicals that are of pharmaceutical interest are waiting to be produced by the benefits of genetic engineering of microbial synthesis on an industrial scale. In terms of sustainability, combined with economic, environmental, and short life cycle benefits, hetero- and autotrophic microalgae may reach this goal.

## Author Contributions

SJ: conceptualization, writing–original draft preparation, writing-reviewing, editing, and investigation–data collection. CP: conceptualization, writing–original draft preparation, writing–reviewing, editing, and supervision. Both authors contributed to the article and approved the submitted version.

## Conflict of Interest

The authors declare that the research was conducted in the absence of any commercial or financial relationships that could be construed as a potential conflict of interest.

## Footnotes

1 https://efsa.onlinelibrary.wiley.com/doi/abs/10.2903/j.efsa.2006.374
